# Unexpected Presentation of Tracheoesophageal Fistula During Intubation in a Pediatric Patient

**DOI:** 10.7759/cureus.50761

**Published:** 2023-12-19

**Authors:** Margarida Telo, Larissa Morais, Rodrigo Ferreira, Adelaide P Coelho, Ivanete Peixer

**Affiliations:** 1 Anesthesiology, Hospital da Luz Lisboa, Lisbon, PRT; 2 Anesthesiology, Hospital Professor Doutor Fernando Fonseca, Lisbon, PRT; 3 Anesthesiology, Centro Hospitalar Universitário de Lisboa Central - Hospital Dona Estefânia, Lisbon, PRT

**Keywords:** pediatric anesthesiology, button battery ingestion, one lung ventilation, critical airway, acquired tracheoesophageal fistula

## Abstract

Tracheoesophageal fistula (TEF) is an abnormal connection between the trachea and esophagus. This report presents a rare case of a pediatric patient who developed a TEF due to battery ingestion, which was diagnosed during intubation and resulted in cardiac arrest.

A 4-year-old child with a two-year history of battery ingestion presented with severe dehydration, weight loss, and recurrent respiratory tract infections. Chest X-ray revealed a radiopaque foreign body in the esophagus. During general anesthesia for central venous line insertion and after endotracheal intubation, some difficulties in ventilation occurred, characterized by the inability to reach tidal volume, absence of capnography, and stomach distention which led to hypoxia and ultimately to cardiac arrest. Prompt resuscitation (CPR) was initiated, and selective right bronchial intubation during CPR improved the patient's condition. Subsequent bronchofibroscopy performed in the ICU confirmed the TEF, which was surgically corrected during the hospital stay.

TEF poses challenges in anesthesia and airway management, particularly when positive pressure ventilation is used. In this case, the TEF was diagnosed during intubation, highlighting the critical role of clinical expertise and prompt intervention in managing this unexpected pediatric critical event.

## Introduction

A tracheoesophageal fistula (TEF) is an abnormal connection between the trachea and the esophagus. Typically, congenital TEF occurs due to the improper development of the lung bud during embryonic growth, resulting in the separation of the foregut into the esophagus and trachea [1]. Acquired TEF, on the other hand, is relatively rare, especially in the pediatric population [2]. It can develop from various causes, such as malignancy, necrosis associated with prolonged intubation, trauma, infections, and caustics or foreign object ingestion [3].

Battery ingestion has recently increased in incidence and presentation may range from subclinical to severe respiratory distress [1]. This case report presents a unique case of a 4-year-old patient with an atypical presentation of TEF secondary to battery ingestion, with a delayed diagnosis that occurred during the process of endotracheal intubation and led to a critical event of cardiac arrest. This case was previously discussed at Euroanesthesia Congress on December 18, 2021.

## Case presentation

**Figure 1:**
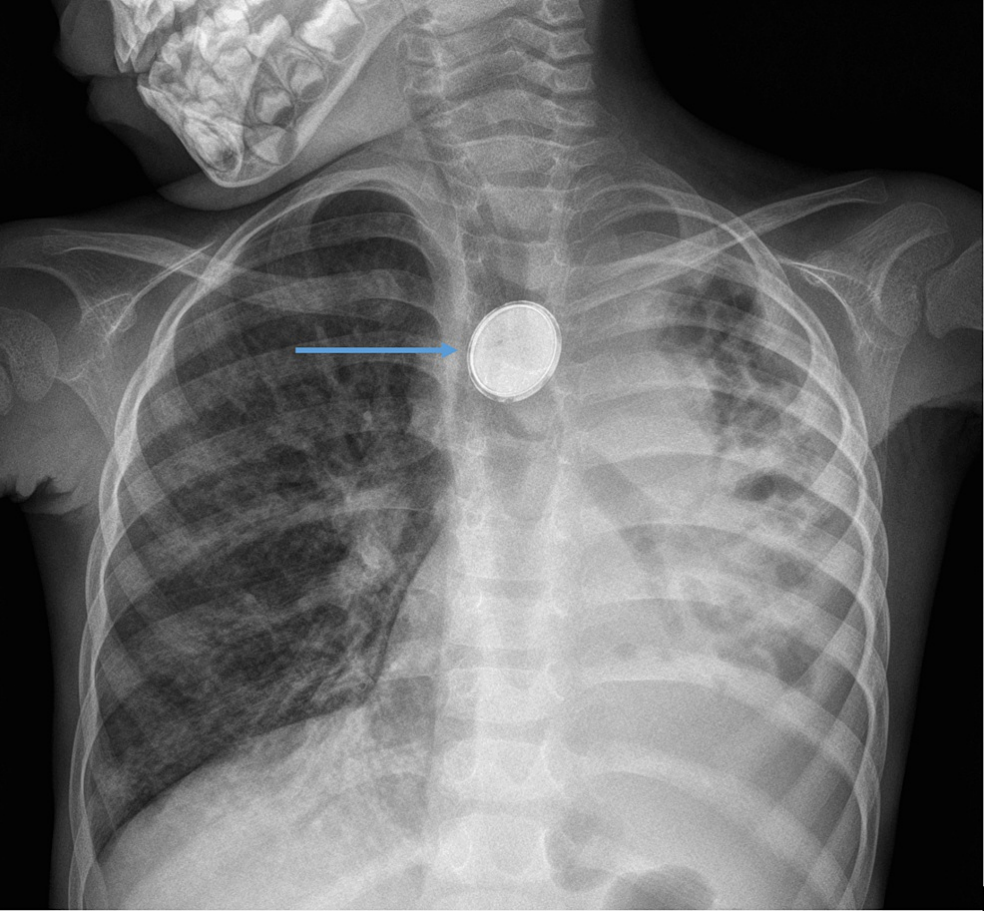
Chest X-ray at admission (anterior-posterior view) Chest radiograph at admission showing the button battery lodged at the esophagus (blue arrow) with the characteristic *Halo sign*.

A 4-year-old child with a suspected two-year history of battery ingestion, with significant weight loss and recurrent respiratory tract infections, was transferred from a low-income country (Guinea-Bissau) to Portugal, presenting with severe dehydration and malnutrition. Chest X-ray (Figure 1) showed left lung heterogeneous opacities and a radiopaque foreign body in the esophagus. To clarify the findings on the X-ray, a chest computed tomography (CT) scan was ordered, but it could not be completed due to the patient's respiratory distress and persistent cough. Also, the patient had poor peripheral venous access and a prolonged course of antibiotics was planned, therefore the child was scheduled for a central venous line insertion under general anesthesia.

**Figure 2:**
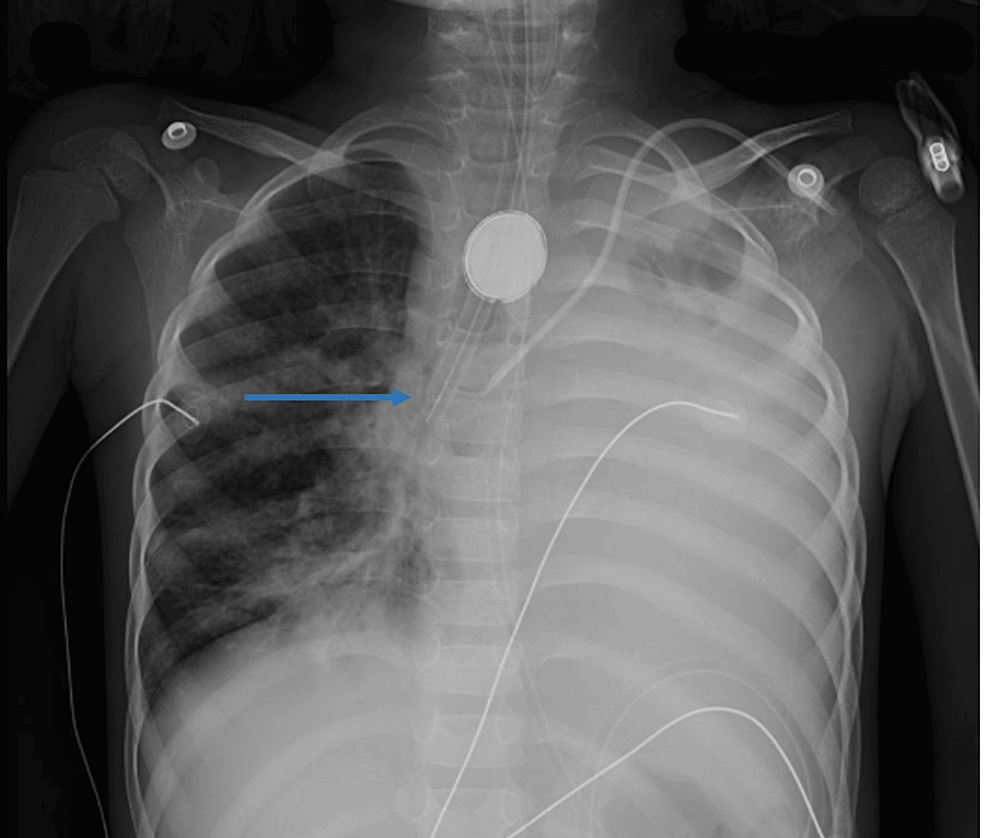
Chest X-ray after intubation Chest radiograph taken after selective right intubation. The blue arrow points to the endotracheal tube in the right bronchus.

The patient presented at the operating room somnolent but arousable and following simple commands. She showed signs of dehydration, respiratory distress requiring oxygen therapy, and malnourishment weighing 8 kg. Regarding aspiration risk and respiratory distress, general anesthesia with rapid sequence induction and orotracheal intubation was chosen. Standard monitoring was applied and after preoxygenation, general anesthesia was induced with 20 mg (2.5 mg/kg) ketamine IV, 10 mg (~1 mg/kg) propofol IV, and 10 mg (1.2 mg/kg) rocuronium IV. After the loss of conscience, a video laryngoscopy with perfect visualization of the cords and intubation with a 4.5 mm cuffed endotracheal tube (ETT) was performed. However, after connecting the patient to the ventilator, there was no thoracic expansion, no capnography curve, and stomach distension was observed. A new video laryngoscopy was performed, correct ETT’s position was confirmed, excluding esophageal intubation. Despite an increase in the fraction of inspired oxygen (FiO_2_) and high ventilatory pressures, ventilation remained very difficult and the end-tidal CO_2_ (EtCO_2_) was absent. The oxygen saturation dropped quickly, and she went into cardiac arrest. Resuscitation was promptly started. During CPR, the presence of a TEF was suspected, so selective intubation to the right bronchus was decided (Figure 2), which allowed clinical improvement and the patient recovered after the second cycle of CPR. One-lung ventilation resulted in the presence of capnography with high EtCO_2_ (80 mmHg), a rise in oxygen saturation, and patient stabilization. Reversal of the neuromuscular blockade to restore spontaneous ventilation was performed with 16 mg/kg of sugammadex.

**Figure 3:**
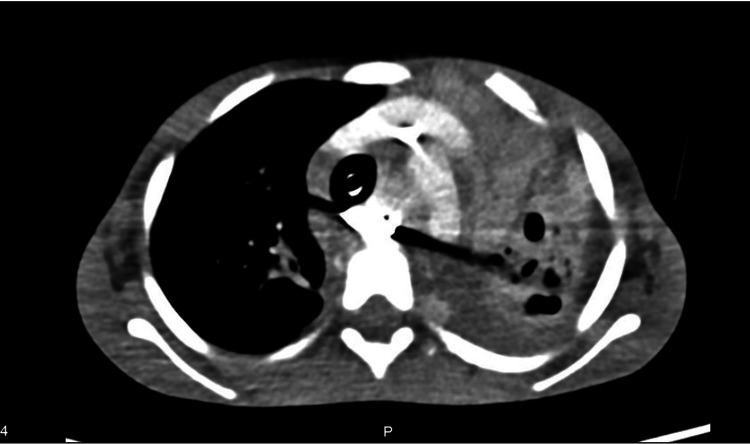
Axial CT scan of the chest after intubation CT scan with a foreign body impacted in the medium third of the esophagus, signs of chronic mediastinitis, chronic left lung atelectasis, and bronchiectasis.

**Figure 4:**
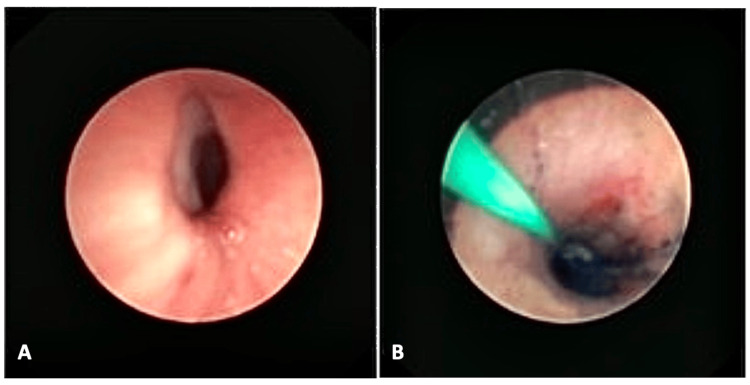
Diagnostic bronchofibroscopy performed in the ICU (A) Left main bronchus with 40% lumen reduction by internal compression. The TEF site was not directly visualized, although there was granulation at this point. (B) The presence of methylene blue in the endotracheal tube, carina, and both bronchus, after injection through the nasogastric tube (25 mL), confirmed the presence of TEF.

The patient was then transported ventilated to the intensive care unit. The chest CT scan (Figure 3) showed a foreign body impacted in the proximal esophagus, signs of mediastinitis and left lung atelectasis and bronchiectasis. The diagnosis of TEF was documented by bronchofibroscopy (Figure 4) with a methylene blue leak test, although the fistula opening was not visualized.

During her hospital stay, she was submitted to a surgical TEF correction with a bovine pericardium patch (PERI-GUARD, *Baxter*) and extraction of a CR2032 battery, without complications

## Discussion

The inadvertent swallowing of disc batteries has become a growing concern in the pediatric population due to the widespread use of electronic devices [3]. Button batteries have a relatively large and flat surface area so they are more likely to become lodged in the esophagus [4]. Normally, the location is in the thoracic inlet (60-80%) but they can also become lodged in the gastroesophageal junction (10-20%), and at the level of the aortic arch (5-20%) [3].

The battery involved in this case was a 3-volt CR2032 lithium battery which is one of the most common button battery types and is associated with the most severe complications. Although the mechanism of tissue injury of lithium batteries is multifactorial, animal studies suggest that damage arises mostly from the contact of both poles of the battery with the esophageal mucosa, generating an electrical current that results in electrolysis, an increase in tissue pH, and an alkaline reaction causing esophageal tissue liquefaction necrosis. Depending on the duration and location of the battery, this lesion may extend beyond the esophageal mucosa into deeper esophageal tissue and adjacent organs and can lead to tracheal perforation and emphysema, pneumomediastinum, mediastinitis, or TEF formation [4].

As presented in this case, acquired TEF is frequently misdiagnosed [5]. Most patients are asymptomatic and even when symptoms are reported, they are often nonspecific and mistakenly associated with more common illnesses, such as pneumonia [6]. Also, the time of battery ingestion is often unknown because it involves unwitnessed ingestions. This patient had a history of ingestion two years before and the only symptomatology was recurrent respiratory infections and frequent bouts of coughing while eating or drinking (also known as Ohno’s sign) [5].

Although there are many methods for diagnosing TEF, diagnosis can be difficult. Radiographic contrast studies in children may not be easy because of technical difficulties and the risk of pulmonary aspiration [6]. A plain radiograph is useful to determine the battery’s size and location. Usually, button batteries have a typical double ring or “halo sign”, in contrast to coins [4]. Multislice CT has the advantage of scanning a large thoracic volume in a single breath-hold without contrast media [6]. When inconclusive, a CT virtual bronchoscopy can be an option to help locate the battery and check for complications. In cases with TEF, an invasive bronchoscopic examination is the gold standard to establish the level of fistula and distance from the carina [7].

In this case, the only exam available before inducing general anesthesia was the plain chest radiograph, revealing a battery located in the proximal esophagus. The negative pole can be identified as the narrow side of the battery on the lateral radiograph where an increase in local pH and tissue necrosis is most likely to occur [4]. If a lateral view had been accessible, we could have observed that the negative pole was oriented anteriorly toward the adjacent trachea. This information could have alerted the team to a higher risk of the presence of a TEF.

Airway and ventilation management in the presence of TEF is challenging and spontaneous ventilation should be maintained whenever possible [8,9]. In this case, we chose general anesthesia with rapid sequence induction and orotracheal intubation, regarding the risk of aspiration and respiratory distress. Therefore, we observed the consequences of applying positive pressure ventilation in the presence of a large TEF: a significant air leak, difficulty in delivering the target tidal volumes, and esophageal and gastric insufflation, increasing an inherent risk of aspiration and potential pneumonitis [10]. Recognition of these features was the key to diagnosis of TEF which triggered the decision to revert the neuromuscular block to restore spontaneous ventilation and to reposition the ETT. As the initial placement of the ETT above the fistula failed and assuming that the fistula involved the left mainstem bronchus, because of the left lung opacities on the chest X-ray, we concluded that the only way to ventilate the patient was through selective intubation of the right mainstem bronchus.

The main learning point from this case is that TEF is a known complication of battery ingestion and clinicians should bear that in mind when managing the airway of these patients. In this case, the confirmation of battery swallowing in the X-ray, the long time between the suspected ingestion and hospital admission (two years), the history of recurrent pneumonia and anorexia, and the opacities on the X-ray should have drawn the attention of the team to the possibility of TEF's presence. If so, sedation with maintenance of spontaneous breathing would have been a better anesthetic choice and this critical event could have been prevented.

## Conclusions

In conclusion, this case, which is unique in the literature, highlights the challenges in diagnosing and managing a TEF caused by battery ingestion in a pediatric patient, during general anesthesia and using positive pressure ventilation. Differential diagnosis of hypoxemia and lack of EtCO_2_ after correct intubation permitted the presumption of a TEF and resolution of the problem with the right endobronchial intubation.
